# Fruits and Berries

**Published:** 1871-12

**Authors:** 


### FRUITS AND BERRIES

Ought to be cultivated on every farm as a means of comfort, luxury, and health; they could be made a source of large profit. If the people would eat half the amount of meats they now do, and more than double the amount of fruits and berries, the public health would be greatly increased, and a large share of ordinary diseases prevented.

A single apple-tree has yielded four thousand apples. A good apple is a great luxury in a large city, and readily sells on the street for five cents; they easily bring from six to ten dollars a barrel. If a man were to set out half an acre of apple-trees, each tree forty feet from its neighbor, and trimmed so that the highest twig could be reached by standing on a chair, a little mine of wealth would be the result. The advantages of this low trimming are numerous: the ground would be kept shaded around the tree, and thus abundant moisture would always be afforded to the surface roots; the fruit could be picked by the hand without being bruised; and even if it fell, but little injury would be done; the trunk and limbs would grow stronger, with less liability to break down by a large crop; the tree itself could be more easily and perfectly trimmed, so as to allow the sun to get in among the fruit, and also the air for ventilation and the summer showers for growth; while the trunk itself would be protected from the burning summer sun.

A farmer who had a few acres to spare might turn them over to his children, and make their culture a source of profit, of pleasure, and of health. Seeing that they could make money by it, both boys and girls would prefer work to play, and with very little parental encouragement would sell their crops, save up their money, and thus themselves be saved for life; for it is a fact, that when once a boy or girl gets a taste of saving money, and is shown how it may be put out at interest and thus accumulate without further work, a business habit is fixed, a disposition to economize, to not draw out any money unless it is really proper and needful; and thus is implanted early the germ of a successful business life.

One of the first steps in a programme like this is to have some idea of space, of room, of measurement of acres. Various shaped acres can be laid out thus: —

| | | | | |
|---|---|---|---|---|
| 5 | yards wide, by | 968 | long, contain | one acre. |
| 10 | " | 484 | " | " |
| 20 | " | 242 | " | " |
| 40 | " | 121 | " | " |
| 99 | feet wide, by | 440 | " | " |
| 110 | " | 396 | " | " |
| 198 | " | 220 | " | " |

To make fruit trees bear largely for many years in succession, the soil under them should not be cultivated, should be ploughed summer and fall, and manured every year; it is said that the best manure for bushes and trees is their own leaves and twigs; these should be gathered in a heap every autumn, mixing therewith some barnyard manure, to be dampened occasionally, to keep the leaves from getting dry and being blown away; then spread out in the early spring; in addition, a bushel of lime mixed well with two or three bushels of wood ashes, spread under each tree once a year, would add greatly to their fruitfulness.

The reason fruit trees die in a few years, or begin to bear less fruit and of an inferior quality, is because the trees, not being manured, exhaust the quality of the soil about them, necessary for their sustenance. A most instructive proof of this is found in a section or slice of a California tree which has been on exhibition near the Central Park during the past summer.

It is known that if a tree is cut down and sawn across, its age is determined by the number of rings which are counted; each ring stands for a year, as each rattle of a snake counts for one year to its possessor. There were counted in this tree twenty-three hundred rings, from the centre outwards; the first were very clear and distinct, while those approaching the outer circumference were so small and so close together as to become almost indistinguishable; this is clear proof that this giant of the forest was

TWENTY-THREE HUNDRED YEARS OLD.

When the great Cæsar was a child, this same tree was four hundred years old; when the prophet Malachi was a boy, this tree was living, and probably making its first ring. This is rather a digression, but the point in connection is this, that if a tree is not manured, it exhausts in time the element in the soil which nourishes it, and finally it grows very slowly; thus in the case of the California tree, in the course of ages, the element which was at first afforded in great abundance gave a large supply of food, and the growth was rapid, perhaps an eighth of an inch a year, but in 1860 it was not much beyond a hair's breadth; showing that trees to be vigorous and thrifty, and grow fast, and be highly productive, must be manured, like the cornfield and the garden; and unless this is done, will die prematurely of starvation.

